# Silibinin inhibits aberrant lipid metabolism, proliferation and emergence of androgen-independence in prostate cancer cells via primarily targeting the sterol response element binding protein 1

**DOI:** 10.18632/oncotarget.2488

**Published:** 2014-09-16

**Authors:** Dhanya K. Nambiar, Gagan Deep, Rana P. Singh, Chapla Agarwal, Rajesh Agarwal

**Affiliations:** ^1^ Department of Pharmaceutical Sciences, Skaggs School of Pharmacy and Pharmaceutical Sciences, University of Colorado Anschutz Medical Campus, Aurora, CO, USA; ^2^ School of Life Sciences, Jawaharlal Nehru University, India; ^3^ University of Colorado Cancer Center, Aurora, CO, USA

**Keywords:** Prostate cancer, lipogenesis, chemoprevention, phytochemicals, AMPK, SREBP1

## Abstract

Prostate cancer (PCA) kills thousands of men every year, demanding additional approaches to better understand and target this malignancy. Recently, critical role of aberrant lipogenesis is highlighted in prostate carcinogenesis, offering a unique opportunity to target it to reduce PCA. Here, we evaluated efficacy and associated mechanisms of silibinin in inhibiting lipid metabolism in PCA cells. At physiologically achievable levels in human, silibinin strongly reduced lipid and cholesterol accumulation specifically in human PCA cells but not in non-neoplastic prostate epithelial PWR-1E cells. Silibinin also decreased nuclear protein levels of sterol regulatory element binding protein 1 and 2 (SREBP1/2) and their target genes only in PCA cells. Mechanistically, silibinin activated AMPK, thereby increasing SREBP1 phosphorylation and inhibiting its nuclear translocation; AMPK inhibition reversed silibinin-mediated decrease in nuclear SREBP1 and lipid accumulation. Additionally, specific SREBP inhibitor fatostatin and stable overexpression of SREBP1 further confirmed the central role of SREBP1 in silibinin-mediated inhibition of PCA cell proliferation and lipid accumulation and cell cycle arrest. Importantly, silibinin also inhibited synthetic androgen R1881-induced lipid accumulation and completely abrogated the development of androgen-independent LNCaP cell clones *via* targeting SREBP1/2. Together, these mechanistic studies suggest that silibinin would be effective against PCA by targeting critical aberrant lipogenesis.

## INTRODUCTION

Dysregulated metabolism is now accepted as one of the hallmarks of cancer [1], and metabolic alterations supporting high growth rate and energy requirements are essential for the sustained tumor growth and progression. The initial observations showcasing the fact that tumor cells are highly glycolytic in nature, led to studies focusing on metabolic variations as a target for cancer therapy [2]. Glucose metabolism henceforth has been extensively studied; however, in recent years, studies have recognized that the metabolic rewiring aiding cancer growth are not just limited to glucose metabolism but also involved other metabolic fluxes including lipid and amino acids [3, 4]. The importance of lipids in cancer progression was established after several studies showing that normal cells, excluding liver and adipose, meet their requirement of lipids through uptake of free fatty acids from the diet; however, in case of cancer cells, more than 90% of their elevated lipid needs, are fulfilled by *de novo* lipogenesis [5-7]. Regarding prostate cancer (PCA), several studies have shown that its precursor lesions undergo exacerbated endogenous lipogenesis, irrespective of extracellular or circulating lipids levels [6-8]. The higher *de novo* lipogenesis in PCA cells has been linked with their increased demand for membranes, energy storage, redox balance, protection from cell death, and activation of several intracellular signaling pathways during uncontrolled cellular proliferation [6-9]. Besides, during androgen deprivation therapy, lipids (cholesterol) play an important role in the *de novo* synthesis of androgens by PCA cells, providing them self-sufficiency in androgen receptor (AR) signaling and hormone-refractory progression [10, 11]. This unique dependence of PCA cells on lipids for their growth and progression provides an excellent opportunity to reduce PCA burden *via* inhibiting lipogenesis and associated molecular regulators using non-toxic small molecules. Silibinin, isolated from the seeds of milk thistle (*Silybum marianum*) plant, is widely consumed as a hepatoprotective agent and has shown strong efficacy against PCA both as anti-cancer and chemopreventive agent in various cell culture and animal models, and is currently being investigated for its beneficial effects in PCA patients [12-17]. Accordingly, in the present study, employing both PCA and non-neoplastic prostate epithelial cells, for the first time we examined the detailed effect of silibinin on cellular lipid levels as well as molecular regulators of lipogenesis.

In PCA cells, glucose-derived carbons are shunted from the mitochondrial TCA cycle (as citric acid) to cytosol for lipid synthesis. Importantly, expression and activity of enzymes involved in lipid synthesis such as ATP-citrate lyase (ACLY), acetyl Co-A carboxylase (ACC), fatty acid synthase (FASN), and stearoyl-CoA desaturase 1 (SCD1) are upregulated and play an important role in PCA [6, 7, 9, 18, 19]. Furthermore, the expression of master transcriptional regulator of lipid synthesis enzymes, sterol regulatory element binding protein 1 (SREBP1), is strongly correlated with Gleason grade [7, 20]. SREBP1 overexpression alone is sufficient to increase tumorigenicity and invasiveness of PCA cells, while its inhibition decreases *de novo* fatty acid synthesis and causes PCA growth inhibition and apoptosis induction [6, 20]. SREBP1 is also the critical link between oncogenic signaling and tumor metabolism [7]. For example, Akt and mTORC1 promote nuclear accumulation of mature SREBP1, and in turn Akt/mTORC1 signaling is activated by SREBP1-mediated lipogenesis [21]. Similarly, a negative regulator of mTOR pathway, AMP-activated protein kinase (AMPK) is reported to phosphorylate SREBP1 and prevent its proteolytic activation [6, 8]. Our extensively published studies have shown that silibinin targets various components of oncogenic signaling in a panel of human and mouse PCA cells and animal models [22-26]; however, silibinin effect on SREBP1 expression as well as its role in the anti-cancer efficacy of silibinin have not been examined yet. Results from present study showed that silibinin effectively decreases SREBP1 expression through AMPK activation in PCA cells, and that silibinin-mediated SREBP1 inhibition is critical for its anti-cancer efficacy against PCA. Since lipid synthesis in PCA cells is controlled by androgens, and under low androgen conditions, lipogenesis regulators play an important role in androgen biosynthesis [27, 28], we also examined silibinin effect on androgen-induced lipid accumulation as well as lipogenesis regulators (SREBP1/2) expression under low androgen conditions. Our results showed that silibinin treatment strongly inhibited the synthetic androgen R1881-induced lipid accumulation as well as completely abrogated the development of androgen-independent clones via targeting SREBP1/2 expression under low androgen condition.

## RESULTS

### Human PCA cells exhibit lipogenic phenotype

**Figure 1:**
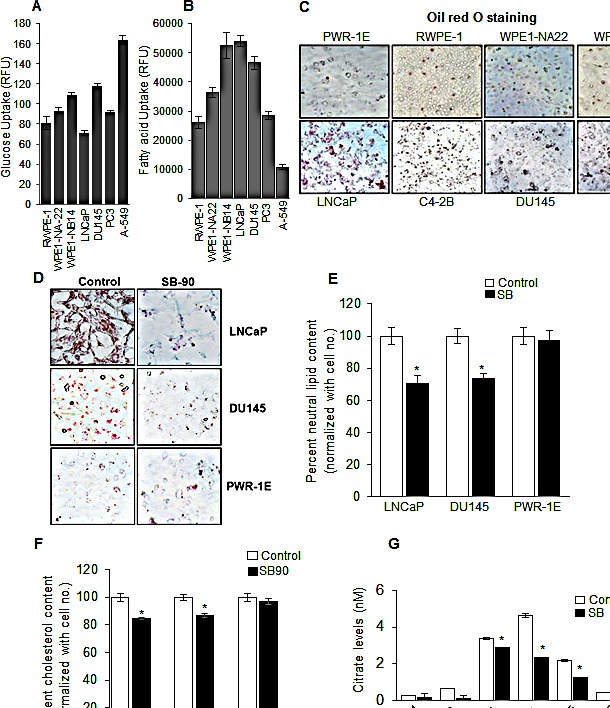
PCA cells exhibit a lipogenic phenotype, and silibinin inhibits neutral lipids, free cholesterol and citrate levels selectively in human PCA cells In each case, equal number of cells were plated and analyzed for (A) glucose uptake, (B) fatty acid uptake, and (C) neutral lipids by ORO staining following procedures detailed in the ‘Materials and Methods’. (D-E) Photomicrographs (at 200x) and quantification of ORO staining in human PCA cells (LNCaP and DU145) and normal prostate epithelial PWR-1E cells following silibinin (90 μM) treatment for 48 h. Quantification data shown for ORO staining was normalized with respective cell number for each group. (F) Percent cholesterol content measured by Filipin III staining following silibinin (90 μM) treatment for 48 h in LNCaP, DU145 and PWR-1E, cells. Quantification data shown for cholesterol content was normalized with respective cell number for each group. (G) Cells were treated with DMSO or silibinin (90 μM) for 48 h; cell lysates were prepared and analyzed for intracellular citrate levels. *, *p*≤0.001. Abbreviations: SB: Silibinin, RFU: Relative fluorescence units.

In order to understand how PCA cells are unique in terms of their metabolic profile, we first evaluated a series of prostate/PCA cell lines for their glucose and fat uptake rates as well as endogenous lipid levels. We selected non-neoplastic benign human prostate RWPE-1, and neoplastic cells (WPE1-NA22 and WPE1-NB14) derived from RWPE-1 [29], and a panel of human PCA cell lines (androgen dependent LNCaP as well as androgen-independent DU145 and PC3 cells), and also included non-small cell lung carcinoma (NSCLC) A549 cells for comparison. As shown in Figure 1A, prostate/PCA cell lines did uptake glucose that was dependent upon their individual cell growth rate in culture; however, there was no clear trend correlating glucose consumption with aggressiveness of these cell lines, e.g. glucose consumption between non-neoplastic RWPE-1 and prostate adenocarcinoma PC3 cells was almost similar (Figure 1A). Interestingly, glucose uptake by prostate/PCA cells was much lower when compared with NSCLC A549 cells (Figure 1A), suggesting their relatively lesser dependence on glucose metabolism.

Next, we assessed the uptake of fatty acids by these cell lines and to our surprise, all prostate cell lines showed much higher fatty acid uptake compared to A549 cells (Figure 1B). Also, there was some trend in terms of higher fatty acid uptake correlating with cells' aggressiveness, e.g. fatty acid uptake trend of WPE1-NB14 > WPE1-NA22 >RWPE-1 matches with their aggressiveness (Figure 1B). On the contrary, LNCaP cells showed higher fatty acid uptake compared with more aggressive DU145 and PC3 cells, which might be related with the functional AR signaling in LNCaP cells (Figure 1B). Together, glucose and fatty acid uptake data underline the dependence of prostate cells on fatty acid precursors compared to A549 cells, which are more glycolytic in nature.

Next, we assessed the neutral lipid content of various prostate/PCA cells by Oil red O (ORO) staining that mainly stains cellular cholesteryl esters and neutral triglycerides. In this assay, we included non-neoplastic immortalized PWR-1E cells along with other cell lines (RWPE-1, WPE1-NA22, WPE1-NB14, LNCaP, DU145 and PC3). We observed higher ORO staining in all PCA cells, with LNCaP cells showing highest lipid accumulation, while least staining was observed in non-neoplastic PWR-1E and RWPE-1 cells (Figure 1C). This data supported the notion that PCA cells have higher content of neutral lipids.

### Silibinin specifically inhibits neutral lipid, cholesterol and citrate levels in PCA cells

Since we observed that PCA cells possess higher turnover rates of lipid and cholesterol discussed above, we next chose to assess whether silibinin treatment could affect the lipid content in these cells. As shown in Figure 1D and 1E, silibinin treatment significantly reduced the neutral lipid level in PCA LNCaP and DU145 cells but not in non-neoplastic PWR-1E cells. Since ORO dye stains for cholesteryl esters, we next assessed the effect of silibinin on unesterified cholesterol by Filipin III staining. Similar to the results obtained with neutral lipids, we observed a significant decrease in the unesterified cholesterol following silibinin treatment in both LNCaP and DU145 cells but not in PWR-1E cells (Figure 1F).

Normal prostate cells are also known to produce and secrete high amount of citrate into the seminal fluid; however, PCA cells overexpress ACLY, an enzyme that converts citrate to acetyl CoA, which is the substrate for fatty acid biosynthesis. ACLY-catalyzed reaction also generates energy (NADPH) required for fatty acid synthesis. Therefore, next, we measured the level of intracellular citrate in prostate/PCA cells. We observed that the cancer cells especially WPE1-NB14, LNCaP and DU145 have higher level of intracellular citrate (Figure 1G), corresponding to higher lipid accumulation in these cells (Figure 1C), and importantly, silibinin treatment significantly reduced the citrate levels in WPE1-NB14, LNCaP and DU145 cells (Figure 1G).

### Silibinin decreases nuclear SREBP1 level *via* inhibiting its proteolytic activation, and thus down-regulates SREBPs target genes involved in fatty acid and cholesterol metabolism in PCA cells

**Figure 2:**
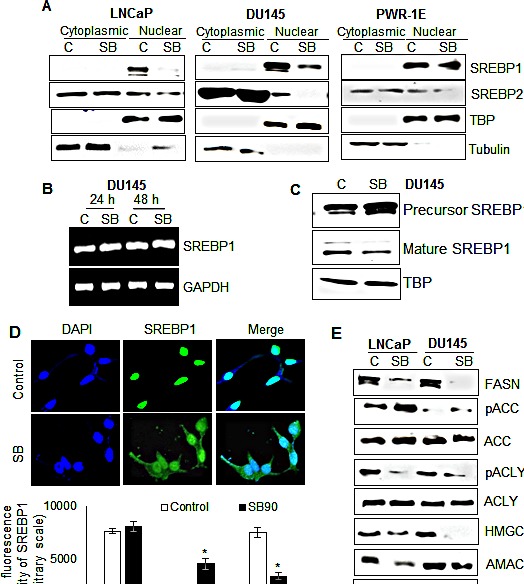
Silibinin decreases SREBP1/2 nuclear localization specifically in PCA cells, and modulates expression/phosphorylation of key molecules involved in lipogenesis (A) LNCaP, DU145 and PWR-1E cells were treated with silibinin (90 μM) for 24 h, and nuclear and cytoplasmic fractions were prepared and analyzed for SREBP1 and SREBP2 expression by Western blotting. Membranes were stripped and reprobed for TBP and tubulin as nuclear and cytoplasmic loading controls, respectively. (B) RT-PCR analysis showing mRNA expression of SREBP1 after treatment with silibinin (90 μM) for 24 and 48 h. GAPDH was used as loading control. (C) Western blot for precursor SREBP1 (125 kDa) and mature SREBP1 (68 kDa) in DU145 cells after treatment with silibinin (90 μM) for 24 h. TBP was used as loading control. (D) Confocal images (upper panel) and quantitative fluorescence intensity (lower panel) for SREBP1 (green-AlexaFluor 488) in LNCaP cells after 24 h of treatment with silibinin (90 μM); DAPI (in blue) stains nuclei. (E) LNCaP and DU145 cells were treated with silibinin (90 μM) for 48 h, and total cell lysates were prepared and analyzed for the total and/or phosphorylated FASN, ACC, ACLY, HMGCR and AMACR by Western blotting. Actin was used as loading control. *, *p*≤0.001. Abbreviations: SB: Silibinin, TBP: TATA binding protein.

SREBPs regulate the transcriptional activation of several genes involved in fatty acid biosynthesis and cholesterol metabolism; therefore, we next analyzed the effect of silibinin on SREBP1 and 2 expression levels. Silibinin treatment did not significantly alter the total levels of the SREBP1/2 in both LNCaP and DU145 cells (data not shown); however, there was a profound decrease in the nuclear SREBP1 following silibinin treatment in both LNCaP and DU145 cells but not in PWR-1E cells (Figure 2A). Similarly, a decrease in SREBP2 protein level was evident only in the nuclear fractions of LNCaP and DU145 cells but not in PWR-1E cells following silibinin treatment (Figure 2A). The observed decrease in nuclear SREBP1 levels by silibinin could be through its effect on SREBP1 transcription and/or through post-translational modifications. To address that, we first examined silibinin effect on SREBP1 mRNA level after 24 and 48 h of its treatment, but found no effect on SREBP1 mRNA level (Figure 2B).

The mammalian SREBP1 is synthesized as 125 kDa precursor with an N-terminal transcription factor domain. In its inactive form, the precursor SREBP1 is present in a complex with SREBP cleavage-activating protein (SCAP), which in turn is bound with another protein, INSIG, and this complex remains bound to the ER membrane [30]. During starvation or sterol depleted condition, there is a decrease in the SCAP-INSIG interaction and the SCAP-SREBP complex translocates to the Golgi apparatus where two sequential proteolytic cleavage marks the activation of SREBPs [31]. The mature soluble SREBP1 (68 kDa) rapidly translocate to the nucleus and activates the target genes by binding to the sterol response element (SRE) within their promoter regions [32]. Since we did not observe any change in SREBP1 mRNA levels with silibinin treatment, but there was a decrease in nuclear SREBP1 levels, we next examined the SREBP1 precursor level after treatment with silibinin and found that while the precursor SREBP1 level was increased, the mature SREBP1 level was decreased, further validating that silibinin inhibits the proteolytic cleavage of SREBP1 (Figure 2C). Next, to confirm inhibitory effect of silibinin on nuclear SREBP1, we employed confocal microscopy, and as shown in Figure 2D (images and bar diagram), SREBP1 expression was mainly nuclear in untreated PCA cells and silibinin treatment reduced the nuclear SREBP1 level while significantly increasing the cytoplasmic SREBP1 level.

Next, we examined the effect of silibinin on the protein expression of several genes whose expression is controlled transcriptionally by SREBPs. As shown in Figure 2E, silibinin treatment strongly decreased the FASN level in both LNCaP and DU145 cells. ACC is another important enzyme involved in maintaining fatty acid homeostasis by catalyzing the carboxylation of acetyl-CoA to form malonyl-CoA. This step precedes the fatty acid synthesis steps catalyzed by FASN, therefore, ACC is considered an important target in PCA [33]. ACC is inactivated by a phosphorylation at Serine-79 site by AMPK or at Serine-1200 site by protein kinase A [34]. We found that there was a significant up-regulation of ACC phosphorylation at Serine-79 site especially in LNCaP cells by silibinin without any significant change in total ACC level (Figure 2E), suggesting an inactivation of ACC following silibinin treatment. Another key regulator of *de novo* fatty acid synthesis is ACLY [35]. We found that silibinin inhibited the phosphorylation of ACLY at Serine-455 site (Figure 2E), thereby stopping the utilization of citrate for synthesis of acetyl-CoA. 3-hydroxy-3-methyl-glutaryl-CoA reductase (HMGCR) catalyzes the rate limiting step in cholesterol biosynthesis. It is suggested to be an important player in PCA progression, as it is shown to contribute to intra-tumoral androgen synthesis [10]. We found that silibinin strongly decreased the HMGCR level in both LNCaP and DU145 cells (Figure 2E). Another metabolic gene α-methylacyl-CoA racemase (AMACR) is reported to be overexpressed in PCA [36]. It is a peroxisomal and mitochondrial enzyme capable of racemizing the α-carbon of various α-methylacyl-CoA derivatives. We found that silibinin also strongly down-regulated the expression of AMACR especially in LNCaP cells.

### Silibinin regulates SREBP1 expression *via* activating AMPK

**Figure 3:**
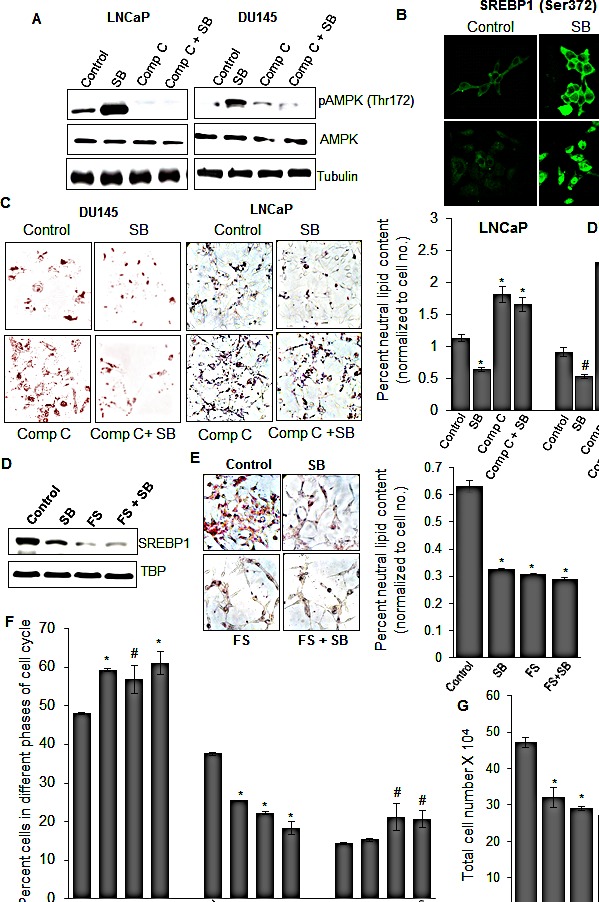
Silibinin decreases SREBP1 expression *via* activating AMPK, and does not show additional efficacy against PCA cells in the presence of SREBP1 inhibitor fatostatin (A) LNCaP and DU145 cells were treated with silibinin (90 μM) with or without compound C (10 μM), and at the end of 12 h, total cell lysates were prepared and analyzed for phosphorylated and total AMPK levels by Western blotting. Membranes were stripped and probed with tubulin antibody to assess protein loading. (B) Confocal microscopy images (at 1000x) showing SREBP1 phosphorylation (Serine-372) following treatment with silibinin (90 μM) for 24 h in LNCaP and DU145 cells. (C) Photomicrographs (at 200x) (left panel) and quantification (right panel) of ORO staining in LNCaP and DU145 cells after 48 h of treatment with silibinin and/or compound C. Quantification data shown for ORO staining was normalized with respective cell number for each group. (D) LNCaP cells were treated with silibinin (90 μM) and/or fatostatin (20 μM), and 24 h later, nuclear lysates were prepared and analyzed for SREBP1 expression by Western blotting. Membranes were stripped and probed with TBP antibody to assess protein loading. LNCaP cells were treated with silibinin (90 μM) and/or fatostatin (20 μM) for 48 h and analyzed for: (E) ORO staining (photomicrographs at 200x, left panel; quantification, right panel), (F) percent of cell in different phases of cell cycle, and (G) total cell number. *, *p*≤0.001; #, *p*≤0.01. Abbreviations: Comp C: Compound C, SB: Silibinin, TBP: TATA binding protein, FS: Fatostatin.

To examine potential mechanisms of silibinin-mediated decrease in SREBP1 expression, we focused on signaling mechanisms that regulate SREBP1 expression at post-translational level. One of the key signaling molecules, known to regulate SREBP1, is AMPK, which is an energy-sensing molecule with serine/threonine kinase activity and is activated in the cells in response to metabolic stress (with a higher AMP/ATP ratio) [37]. Once activated *via* phosphorylation at Threonine-172 site, AMPK inhibits bioenergetic pathways, especially lipogenesis by either direct phosphorylation of ACC or indirectly (*via* targeting SREBP1) to reduce the transcriptional activity of lipogenic genes such as FASN and ACLY [38]. Since we found down-regulation of SREBP1 and its target genes by silibinin, we inferred that this effect could be mediated by up-regulation of AMPK. Indeed, treatment with silibinin resulted in a robust up-regulation of AMPK phosphorylation in LNCaP and DU145 cells at 12 h that was completely inhibited in the presence of AMPK inhibitor, compound C (Figure 3A), prior to a decrease in SREBP1 expression by ~16 h following silibinin treatment (data not shown). Activated AMPK is known to inhibit SREBP1 activation and nuclear translocation by phosphorylating it at Serine-372 site [39], and thus we also employed confocal microscopy to assess whether SREBP1 was phosphorylated by AMPK at Serine-372 site. A prolific increase in the phosphorylated levels of SREBP1 was observed in both LNCaP and DU145 cells in response to silibinin treatment (Figure 3B). Consistent with these findings, next we examined whether inhibiting silibinin-caused AMPK activation would reverse silibinin effect on lipid accumulation. Indeed AMPK inhibitor compound C alone treatment resulted in a significant increase in lipid accumulation in both LNCaP and DU145 cells as measured by ORO staining, and when combined with silibinin, it reversed the inhibitory effect of silibinin on lipid accumulation in these PCA cell lines (Figure 3C, left and right panels).

### Silibinin does not show additional efficacy against PCA cells in the presence of SREBP inhibitor fatostatin

Fatostatin (FS) is a synthetic inhibitor of SREBPs and has been shown to block the activation of SREBPs, thereby decreasing the transcription activity of downstream effectors [40]. Since we observed that silibinin mediates a similar action on SREBPs, we did an analogue experiment to further confirm that silibinin inhibits lipogenesis in PCA cells solely *via* SREBPs inhibition. Fatostatin treatment alone or in combination with silibinin resulted in a decrease in nuclear SREBP1, with combination showing a better decrease compared with either fatostatin or silibinin alone (Figure 3D). However, the decrease in neutral lipid level observed with combination was comparable to either agent alone, where fatostatin alone reduced the lipid content by 48%, silibinin by 51% and there combination by 54%, suggesting that there was no significant additional effect in combination (Figure 3E). Since inhibiting lipid synthesis would essentially deprive the cells of cellular biomolecules for membrane synthesis and other processes important for cell division, we propounded that silibinin-mediated reduction in lipid level would be associated with its (earlier reported) PCA cell growth inhibitory effects [12, 22, 23]. Accordingly, we next examined the effect of silibinin and fatostatin on cell cycle progression and cell proliferation. Fatostatin and silibinin alone or in combination resulted in comparable G1 phase arrest; 56%, 59% and 61% cells in G1 phase compared to 48% in control, respectively (Figure 3F). Similarly, individual treatment of fatostatin or silibinin resulted in 38% and 32% decrease in total cell number and their combination caused 42% decrease in total cell number (Figure 3G). Together, these results validated that both fatostatin and silibinin act by targeting the same molecule, i.e. SREBP1.

### SREBP1 overexpression abrogates anti-PCA effects of silibinin

**Figure 4:**
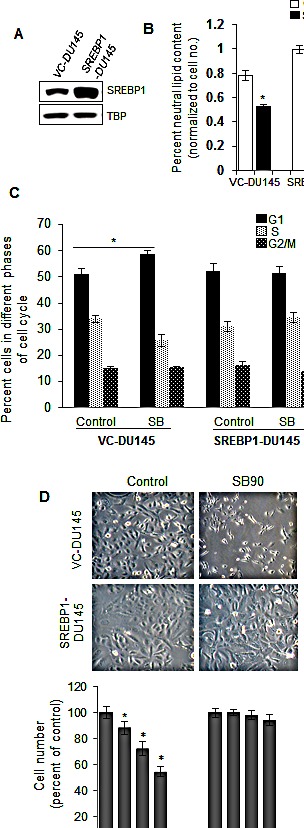
SREBP1 overexpression abrogates silibinin efficacy against PCA cells (A) DU145 cells were transfected with pLX304-SREBP1 plasmid or empty vector using lentiviral transfection system and stable clones were identified through blasticidin-based selection. Western blotting was performed to show nuclear SREBP1 expression in vector control (VC-DU145) and SREBP1 overexpressing DU145 cells (SREBP1-DU145). TBP was used as loading control. (B) Quantification (normalized with respective cell number) of neutral lipid content in VC-DU145 and SREBP1-DU145 cells following treatment with silibinin (90 μM) for 48 h. (C) Cell cycle analysis by saponin-PI method after treatment with silibinin (60 μM) for 48 h in VC-DU145 and SREBP1-DU145 cells. (D) Representative pictures and total cell number quantification after treatment with various doses of silibinin (30-90 μM) for 48 h in VC-DU145 and SREBP1-DU145 cells. *, *p*≤0.001. Abbreviations: SB: Silibinin, TBP: TATA binding protein.

To test directly whether SREBP1 plays an essential role in the down-regulation of lipid accumulation and inhibition of cell growth in PCA cells by silibinin, we transduced DU145 cells with a lentiviral expression vector constitutively driving the expression of SREBP1. After lentiviral infection and selection, positive clones were checked for SREBP1 expression by immunoblot analysis to confirm the transduction and SREBP1 overexpression in SREBP1-DU145 cells compared to vector control-DU145 (VC-DU145) cells (Figure 4A). Next, effect of silibinin treatment was examined on neutral lipid content in both VC-DU145 and SREBP1-DU145 cells. We observed a 22% increase in the lipid content in SREBP1-DU145 cells compared with VC-DU145 cells, but importantly, the induced SREBP1 over-expression abrogated silibinin-mediated inhibitory effect on the lipid content (Figure 4B). The lipid content was reduced by 32% in VC-DU145 by silibinin, but only by 10% in SREBP1-DU145 cells (Figure 4B).

As mentioned above, since silibinin also induces a cytostatic effect in PCA cells by inducing a G1 arrest, we next questioned whether over-expression of SREBP1 would abrogate this effect of silibinin. We found that silibinin treatment of VC-DU145 cells resulted in a G1 arrest (59% cells in G1 phase compared to 51% in DMSO vehicle control, p≤0.001), but this effect of silibinin was compromised in SREBP1-DU145 cells showing no change in the cell cycle distribution (Figure 4C). Next, we compared the effect of silibinin treatment on cell growth in VC-DU145 and SREBP1-DU145 cells. Photomicrographs captured at the end of silibinin treatment clearly showed that it has a strong effect on the morphology and growth of VC-DU145 cells but not SREBP1-DU145 cells (Figure 4D, upper panel). Further quantitative analysis confirmed that whereas silibinin causes a strong and dose-dependent decrease in the cell growth of VC-DU145 cells, there was almost no growth inhibitor effect of silibinin in SREBP1-DU145 cells (Figure 4D, lower panel). Together, these results clearly suggested that silibinin-caused PCA inhibition is mainly *via* inhibiting SREBP1 and lipid accumulation in PCA cells.

### Silibinin inhibits androgen-induced lipid accumulation as well as androgen independent growth of LNCaP cells *via* targeting SREBPs

**Figure 5:**
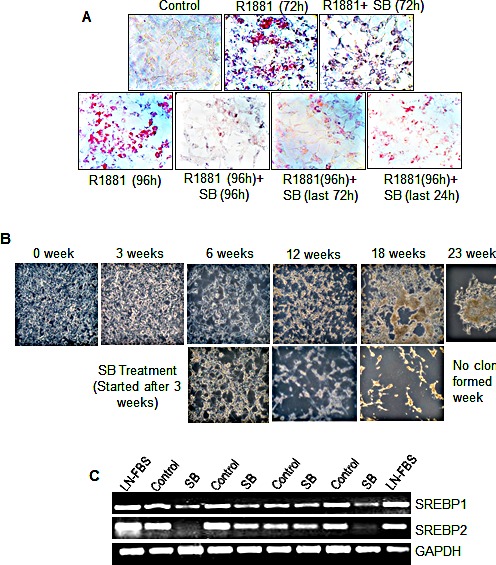
Silibinin inhibits R1881-induced lipid accumulation and androgen independent growth of LNCaP cells *via* decreasing the SREBP1/2 expression (A) LNCaP cells (2×10^5^ per plate) were seeded, and 24 h later, cells were starved for 24 h in 10% cFBS media and then treated daily with R1881 (10 nM) with or without silibinin (90 μM) for 72 or 96 h (upper panel) or induced with R1881 for 96 h and silibinin (90 μM) was added after 24 h or after 72 h (lower panel). At the end of each treatment, cells were fixed and stained with ORO. Representative photomicrographs of ORO staining are shown (at 200x). (B) LNCaP cells were grown to 70% confluency in T-75 flasks under 10% FBS condition, and then transferred to low androgen media (10% cFBS) and maintained till androgen independent clones developed. One set of flask was also treated with 10 μM silibinin, starting from the 3^rd^ week of androgen deprivation. Representative pictures at different time intervals of the experiments are presented. (C) Employing the samples from the experiment shown in panel B, RNA was isolated at different time intervals and RT-PCR was performed to analyze the expression pattern of SREBP1 and SREBP2. GAPDH was used as a loading control. AI-clones of LNCaP cells are labeled as ‘control’ in the gels shown. Abbreviations: SB: Silibinin, GAPDH: Glyceraldehyde 3-phosphate dehydrogenase, AI: Androgen independent, LN-FBS: LNCaP cells grown under 10%FBS condition.

Androgens such as testosterone and dihydrotestosterone (DHT) are known to induce neutral lipid accumulation in LNCaP cells, while androgen antagonist Casodex inhibits lipogenesis [41]. This suggests that lipid accumulation and synthesis in PCA cells is influenced by androgens. In light of the fact that silibinin inhibits AR signaling in PCA cells [42, 43], we also sought to understand whether the inhibitory effect of silibinin on lipid content was mediated *via* its inhibitory effect on androgen-AR signaling. To study this, we stimulated LNCaP cells with 10 nM R1881 under charcoal stripped serum (cFBS) condition. We observed a robust accumulation of lipid in LNCaP cells in response to R1881 treatment, but silibinin addition along with R1881 completely inhibited the R1881-induced neutral lipid accumulation in these cells (Figure 5A). In another experiment, we cultured these cells in media containing R1881 for 96 h and treated them with silibinin after 24 h (i.e. for last 72 h) or after 72 h (i.e. only for last 24 h); we nonetheless found inhibition in lipid accumulation (Figure 5A, lower panel), advocating that silibinin could inhibit the androgen-induced lipid accumulation in PCA cells.

It is now believed that androgen-independent growth of PCA cells also depends upon lipogenesis, as lipids (such as cholesterol) act as precursors for the synthesis of androgens [44]. Accordingly, next we also examined whether silibinin could inhibit the hormone-refractory growth of PCA cells through its inhibitory effect on key lipogenesis regulator SREBP1/2. LNCaP cells were grown to 70% confluency and then shifted to androgen free 10% cFBS containing media and maintained in this media till androgen-independent clones emerged. Androgen withdrawal led to inhibition in cell growth, but significant increase in cell death started only after 3-4 weeks of cFBS treatment. Throughout the study, cells were washed and fresh media was added every 3^rd^ day. In the control group, after 18 weeks of androgen withdrawal, we found development and re-growth of androgen-independent clones; however, in the flasks with 10 μM silibinin treatment, there was no re-growth or appearance of colonies even after 23^rd^ week (Figure 5B), which suggests that silibinin inhibited the attainment of androgen-independent phenotype. RNA analysis of LNCaP cells collected at different time-points following androgen withdrawal, showed that SREBP1 and SREBP2 expression did not decrease much and/or remained higher even under androgen deprived conditions, but silibinin treatment reduced their expression at all the time-points (Figure 5C). This finding further supported our hypothesis that silibinin treatment specifically inhibited SREBP1 and SREBP2, which are important for the androgen-independent growth of these LNCaP PCA cells.

## DISCUSSION

PCA is the most common non-cutaneous cancer in US men and according to the American Cancer Society reports, 233,000 new cases and 29,480 deaths from PCA are estimated in the United States in 2014 [45]. Although curative measures exist for PCA management, significant toxicity is associated with them and thousands of patients succumb to this deadly malignancy every year. Clearly, additional approaches and strategies are needed to better understand and target PCA. One such unique opportunity is to target aberrant lipogenesis in PCA, because a plethora of studies in recent years have shown that this metabolic feature is essential for PCA proliferation, survival, bioenergetics, chemoresistance, etc. [6-9, 19, 20, 46, 47]. However, studies focusing on understanding and targeting aberrant lipid metabolism in PCA cells by non-toxic natural agents are lacking, although a series of such agents have been studied extensively for their PCA efficacy [22, 48]. Results from the present study, for the first time, clearly suggest that natural flavonoid silibinin targets cellular lipid accumulation and, thereby, inhibits PCA cell proliferation.

Prostate tissue is unique in terms of endogenous citrate secretion and turnover. The human prostate gland accumulates and secrets extraordinarily high levels of citrate and zinc in the prostatic fluid (citrate concentration ranges from 40 to 150 mM), which is not found in any other tissues in the body [49]. However, PCA cells, instead of secreting citrate, divert it towards lipogenesis. In PCA cells, citric acid, produced in mitochondrion, is transported to the cytoplasm via citrate-pyruvate shuttle, where it is cleaved by ACLY to generate energy and acetyl CoA, a precursor for lipid biosynthesis. In the present study, we observed higher intracellular citrate level in PCA cells (WPE1-NB14, LNCaP and DU145 cells) compared to non-neoplastic RWPE-1 and less aggressive WPE1-NA22 cells. This suggests that PCA cells utilize citrate for *de novo* lipogenesis, and silibinin treatment reduced the intracellular citrate level via down-regulating ACLY expression. The decrease in intracellular citrate level by silibinin was also well reflected in the inhibition of neutral lipid droplets in PCA cells. Importantly, PCA cells consistently showed a higher lipid level compared with non-neoplastic prostate cells, which is in line with earlier published report showing an aberrant accumulation of esterified cholesterol in lipid droplets of high-grade PCA and metastases [50]. Importantly, the inhibitory effect of silibinin on neutral lipid and cholesterol level was quite specific to PCA cells and no significant change was observed in neutral lipid and cholesterol level in non-neoplastic PWR-1E cells following silibinin treatment. Moreover, a decrease in lipid level by silibinin correlated with its PCA specific growth inhibitory effects reported in this study as well as earlier [51]. This could be explained by the fact that most of the normal cells do not require *de novo* lipogenesis, and therefore, normal cells are less sensitive to inhibition of this pathway by silibinin.

**Figure 6:**
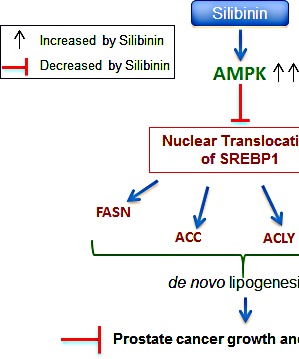
Schematic representation of silibinin-mediated action against lipogenesis and cell growth inhibition in PCA Silibinin treatment activated AMPK, enhanced SREBP1 phosphorylation, decreased SREBP1 nuclear translocation and expression of SREBP1 regulated genes, thereby inhibited lipid accumulation and cell proliferation, and induced cell cycle arrest in PCA cells.

Lipogenesis is mainly controlled by SREBPs, and aberrant activation of SREBPs as well as their target genes has been reported in several cancer types, including PCA [52]. Huang et al have reported that SREBP1 is highly expressed in the nuclei of prostate tumor cells with higher Gleason grades, which correlates with poor prognosis and androgen-independent PCA progression [20]. Results from the present study showed that silibinin decreases the nuclear SREBP1/2 expression specifically in PCA cells. Earlier studies have clearly established that mTORC1 promotes nuclear accumulation of mature SREBP1 [21], while a negative regulator of mTOR pathway, AMPK, physically interacts and phosphorylates SREBP1 (at Ser-372 site) and suppresses its proteolytic cleavage and nuclear translocation [6, 8]. You et al reported that AMPK inhibition leads to activation of SREBP1-mediated lipogenesis [53]. Similarly, AMPK inhibition has been suggested as a causal event in ethanol-induced fatty liver [53]. AMPK activation restricts anabolic pathways including lipogenesis and represses cancer cells growth and induces apoptosis [54-58]. Therefore, we conjectured that silibinin might inhibit SREBP1-mediated lipogenesis through AMPK activation. Indeed, our results showed that silibinin strongly up-regulates AMPK activation in PCA cells, followed by robust inhibitory phosphorylation of SREBP1 at Serine-372 site. AMPK inhibition by compound C also resulted in up-regulation of lipid biosynthesis. This is in line with earlier report by Shi et al that inhibiting AMPK in hepatocytes resulted in lipid droplet accumulation [59]. Importantly, AMPK-mediated SREBP1 inactivation by silibinin seems to be central to its anti-PCA effects (Figure 6), as AMPK inhibitor reversed the inhibitory effect of silibinin on lipid accumulation, and also silibinin did not exert any additional efficacy against PCA cells in the presence of SREBP1 inhibitor fatostatin. Furthermore, growth inhibitory effects of silibinin were completely reversed in PCA cells overexpressing SREBP1. Together, these results suggest that SREBP1 is a novel silibinin target and plays a central role in silibinin efficacy against PCA cells (Figure 6).

*De novo* androgen synthesis and sustained activation of AR signaling are the central events during PCA re-growth under low androgen conditions during hormonal ablation therapy [60-65]. There are several studies suggesting that intra-tumoral androgens could be derived from *de novo* synthesized cholesterol as well as circulating cholesterol from diet [10, 44, 66]. Ettinger et al reported that in LNCaP xenograft model, expression levels of both SREBP1a and SREBP1c were significantly enhanced in mice 2-3 weeks following castration compared to their basal levels [28]. Similar results were also observed in clinical PCA specimens showing an up-regulation of SREBPs and their downstream effector genes during progression to androgen independence [28]. Results from the present study suggested that silibinin could prevent PCA hormone-refractory progression via targeting SREBP1/2 expression. Further studies are however needed in future to confirm silibinin effect on *de novo* synthesis of androgens from cholesterol and resultant activation of AR signaling in PCA cells especially under *in vivo* conditions. In conclusion, our results clearly show that silibinin decreases nuclear level of SREBP1 and lipid accumulation in PCA cells as a novel mechanism of its action against PCA growth and progression. Together, these mechanistic findings suggest that non-toxic silibinin could be useful in PCA prevention and treatment via targeting aberrant lipogenesis.

## MATERIALS AND METHODS

### Cell lines and reagents

All the cell lines were from from ATCC (Manassas, VA). LNCaP, DU145, PC3 and A549 cells were cultured in RPMI1640 media with 10% FBS (fetal bovine serum). PWR-1E, RWPE-1, WPE-NA22 and WPE-NB14 were maintained in keratinocyte growth medium with 5 ng/mL human recombinant epidermal growth factor and 0.05 mg/mL bovine pituitary extract; and as per experimental requirements, these cells were also cultured with 10% FBS or serum free keratinocyte growth medium. Media, serum and other cell culture materials were from Invitrogen-Life Technologies (Carlsbad, CA). Silibinin and fatostatin were from Sigma (St Louis, MO) and dissolved in DMSO as stock solution. The final concentration of DMSO in the culture medium during different treatments did not exceed 0.1% (v/v). Antibodies for SREBP1, HMGCR, and TATA-binding protein (TBP) were obtained from Abcam (Cambridge, MA). Total and/or phosphorylated antibodies for SREBP1, SREBP2, FASN, ACC, ACYL, AMPK, and AMACR were from Cell Signaling Technologies (Danvers, MA). Tubulin antibody was from Santa Cruz Biotechnology (Santa Cruz, CA, USA). Compound C was from Calbiochem (La Jolla, CA). R1881 was from Perkin Elmer (Waltham, MA). ECL detection system and anti-mouse HRP-conjugated secondary antibody were from GE Healthcare (Buckinghamshire, UK).

### Glucose uptake assay

Glucose uptake was measured using the glucose assay-cell based assay kit from Caymen chemicals (Ann Arbor, MI). Cells were seeded at the density of 10,000 cells per well in a 96-well plate and maintained at 37ºC in CO_2_ incubator for 24 h. Next day, the media was removed and replaced with glucose free RPMI. After the end of 6 h, media containing 2-NBDG (150 μg/ml) was added to each well and incubated for 30 minutes in dark. Thereafter, media was removed and processed as per manufacture description. The fluorescence was measured using a plate reader at λ_ex_=485 nm and λ_em_=535 nm.

### Fatty acid uptake assay

Fatty acid uptake was measured using QBT™ fatty acid uptake assay from Molecular devices (Sunnyvale, CA) following manufacturer's protocol. Briefly, cells were seeded at 50,000 cells/well in a 96-well plate. After 24 h of cells seeding, complete media was replaced with serum free RPMI media for 6 h. The cells were treated with 100 μl of 1x loading buffer and transferred to a fluorescent plate reader for kinetic reading every 60 seconds at λ_ex_=485nm and λ_em_=515nm, cutoff 495 nm.

### Citrate measurement

Citrate content in the cells was measured using the citrate colorimetric/fluorometric assay kit from Biovision (Milpitas, CA). Cells were treated with 0.1% DMSO or silibinin (90 μM) for 48 h. Thereafter, cells were homogenized with 100 μl of citrate assay buffer and centrifuged at 16,000 rpm for 10 minutes to remove cell debris. Cell lysates containing equal amount of proteins were used for the deproteinization step which was done with 10 kDa molecular weight cut off spin columns (BioVision, Cat # 1997-25). The samples were then processed as per the manufacturer's protocol, and final concentration of citrate was obtained after extrapolation from the citrate standard curve.

### Oil Red O (ORO) staining

Cells were seeded at 50,000 cells/ well in a 6 well plate. At the end of desired treatment, cells were fixed in 10% buffered formalin for 15 minutes at room temperature, washed twice with distilled water and then with 60% isopropanol for 5 minutes. After isopropanol wash, the plate was completely dried and stained with ORO stain (0.3% ORO in 100% isopropanol, diluted with distilled water in the ratio of 3:2) for 30 minutes. After staining, cells were washed with distilled water to get a clear background. Pictures were captured at 200x magnification under a microscope and lipid content quantitation was carried out by dye elution using 100% isopropanol and absorbance was measured by spectrophotometer at 500 nm.

### Cholesterol estimation

Cells were seeded at the density of 10,000 cells per well in a 96 well plate. After 24 h of cell seeding, complete media was replaced with serum free media with or without silibinin treatment for 48 h. Cholesterol content in cells was detected using cholesterol cell based detection assay kit from Caymen chemicals (Ann Arbor, MI) following manufacturer's protocol.

### Cell growth and cell cycle distribution assays

Cells were plated at a density of 50,000 cells per well in 6-well plate. At the end of desired treatments, total cell number was determined using a hemocytometer. Cell cycle distribution (saponin/PI staining) was analyzed by FACS using University of Colorado Cancer Center Flow Cytometry Core Facility following published method [12].

### Confocal imaging

Cells were grown on cover slips and treated with DMSO or silibinin (90 μM). At the end, cells were fixed in 4% buffered formalin for 30 minutes at room temperature followed by gentle PBS wash three times, and permeabilized with PBST (PBS + 0.3% Triton X-100) for 2 h with gentle shaking; and then blocked for 1 h in CAS block buffer (Invitrogen, 1:1 in PBS). Next, cells were incubated with respective primary antibodies, SREBP1 (1: 500) and pSREBP1 (1: 200) after diluting in PBST (with 1% BSA) overnight with gentle shaking. Thereafter, cells were washed with PBS with 0.1% Triton X-100, and finally cells were incubated with rabbit Alexa-Fluor 488 secondary antibody for 1 h. The coverslips were washed with PBS containing 0.1% Triton X-100 and mounted on slides with ProLong® Gold Antifade Mountant from Invitrogen-Life Technologies (Carlsbad, CA). Images were captured at 1000x magnification on a Nikon inverted confocal microscope using 488/405 nm laser wavelengths to detect SREBP1 or pSREBP1 (green) and DAPI (blue) emissions, respectively. Average fluorescence intensity was quantified using Image J software. In both control and treatment groups, the intensity plot was generated from 20 cells captured from different areas. The corrected cell fluorescence (CF) was obtained after normalizing to the background fluorescence using the formula: CF= Integrated Density - (Area of selected cell × Mean fluorescence of background readings).

### R1881 stimulation and androgen-deprivation growth studies

LNCaP cells (2×10^5^ per plate) were seeded, and 24 h later, cells were starved for 24 h in 10% cFBS media, and then treated daily with R1881 (10 nM in ethanol) with or without silibinin (90 μM) for 3 or 4 days or induced with R1881 for 4 days and silibinin (90 μM) added only for last 72 or 24 h. At the end of each treatment, cells were fixed and stained with ORO. For androgen deprivation studies, LNCaP cells were grown to 70% confluency and then shifted to androgen free 10% cFBS containing media, and maintained in this media till androgen independent clones emerged. Cells were washed and fresh media was added after every 3^rd^ day. In the silibinin treatment group, 10 μM silibinin was added every time to the fresh media. Cells were collected and RNA was isolated from cells at different time-points following androgen withdrawal.

### Reverse transcription-polymerase chain reaction (RT-PCR)

Total RNA was extracted from desired cells using TRIzol® Reagent (Invitrogen-Life Technologies, Carlsbad, CA). cDNA was produced from 1 μg of total RNA by using High capacity cDNA reverse transcription assay kit from Life Technologies. SREBP1 (Forward primer: TCAGCGAGGCGGCTTTGGAGCAG; Reverse primer: TCAGCGAGGCGGCTTTGGAGCAG) and SREBP2 (Forward primer: AACGGTCATTCACCCAGGTC; Reverse primer: GGCTGAAGAATAGGAGTTGCC) mRNA levels were quantified by RT-PCR following standard procedures. GAPDH was used as the loading control.

### Western blotting

Cells were treated with DMSO or silibinin and whole cell or nuclear/cytoplasmic extracts were prepared and immunoblotting was performed following published methods [67]. As needed, 60 μg of protein lysate per sample was denatured in 2x sample buffer and subjected to sodium dodecylsulfate–polyacrylamide gel electrophoresis (SDS-PAGE) on 8-16% Tris–glycine gel (as required based upon the protein molecular weight). The separated proteins were transferred on to nitrocellulose membrane followed by blocking with 5% non-fat milk powder (w/v) in Tris-buffered saline (10 mM Tris–HCl, pH 7.5, 100 mM NaCl, 0.1% Tween 20) for 1 h at room temperature. Membranes were probed for the protein levels of desired molecules using specific primary antibodies followed by the appropriate peroxidase-conjugated secondary antibody and visualized by ECL detection system. As required, membranes were also stripped and re-probed again for protein of interest or with appropriate loading control antibody. The autoradiograms/bands were scanned with Adobe Photoshop 6.0(Adobe Systems, San Jose, CA). In each case, blots were subjected to multiple exposures on the film to make sure that the band density is in the linear range.

### Generation of DU145-vector control and SREBP1 overexpressing stable cell lines

DU145 cells were transfected with lentiviral particles containing pLX304-SREBP1 (Clone ID: ccsbBroad304_06995) or vector control pLX304 (Addgene, Cambridge, MA). Stable transduced cells were selected using blasticidin (10 μg/ml) in complete media for 3-4 weeks. The individual resistant clones were picked and grown separately and maintained in the same selection medium.

### Statistical analysis

All statistical analyses were carried out with Sigma Stat software version 2.03 (Jandel scientific, San Rafael, CA). Mean and SEM were used to describe the quantitative data. One-way ANOVA followed by Tukey's test was used for multiple comparisons and statistically significant difference was considered at p≤0.05.

## Abbreviations

ACC: Acetyl Co-A carboxylase
ACLY: ATP-citrate lyase
AI: Androgen independent
AMACR: α-methylacyl-CoA racemase
AMPK: AMP-activated protein kinase
ANOVA: Analysis of variance
AR: Androgen receptor
DMSO: Dimethyl sulfoxide
ECL: Enhanced chemiluminescence
ER: Endoplasmic reticulum
FASN: Fatty acid synthase
FS: Fatostatin
GAPDH: Glyceraldehyde 3-phosphate dehydrogenase
HMGCR: 3-hydroxy-3-methyl-glutaryl-CoA reductase
mTORC1: Mammalian target of rapamycin complex 1
NADPH: Nicotinamide adenine dinucleotide phosphate
ORO: Oil red O
PCA: Prostate cancer
PI: Propidium iodide
RTPCR: Reverse transcription-polymerase chain reaction
SB: Silibinin
SCAP: SREBP cleavage-activating protein
SCD1: Stearoyl-CoA desaturase 1
SDS-PAGE: Sodium dodecylsulfate–polyacrylamide gel electrophoresis
SEM: Standard error of the mean
SRE: Sterol response element
SREBP: Sterol regulatory element binding protein
TBP: TATA-binding protein
